# Effect of the personality traits of healthy Japanese workers on depressive symptoms and social adaptation, and on the achievement rate of exercise therapy to prevent major depression

**DOI:** 10.3389/fpsyg.2023.1195463

**Published:** 2023-06-21

**Authors:** Atsuko Ikenouchi, Naomichi Okamoto, Tomomi Matsumoto, Reiji Yoshimura

**Affiliations:** ^1^Medical Center for Dementia, Hospital of the University of Occupational and Environmental Health, Japan, Kitakyushu, Japan; ^2^Department of Psychiatry, School of Medicine, University of Occupational and Environmental Health, Japan, Kitakyushu, Japan

**Keywords:** exercise therapy, personality traits, NEO-FFI, healthy worker, prevention of major depression

## Abstract

**Background:**

This study determined the effects of personality traits on depressive symptoms and social adaptation in healthy workers, and the effects of depressive symptoms or social adaptation before and after exercise therapy, and personality traits before exercise therapy on the achievement rates of exercise therapy aimed at preventing major depression.

**Methods:**

Two hundred fifty healthy Japanese workers were given an eight-week walking program as exercise therapy. After excluding 35 participants who had dropped or provided incomplete information, 215 were included in the analysis. The Japanese version of the NEO five-factor inventory was used to assess participants’ personality traits before the exercise therapy. Depressive symptoms were evaluated using the Japanese version of the Zung self-rating depression scale (SDS-J) and social adaptation was evaluated using the Japanese version of the social adaptation self-evaluation scale (SASS-J) before and after the exercise therapy.

**Results:**

The SDS-J scores correlated with neuroticism and negatively correlated with extraversion, agreeableness, and conscientiousness before the exercise therapy. The SDS-J was also negatively correlated with openness in women, but not in men, while the SASS-J was associated with extraversion, openness, agreeableness, and conscientiousness, and negatively correlated with neuroticism. There was no significant change in levels of depression before and after exercise therapy; however, social adaptation increased significantly in men. No association was found between SDS-J and SASS-J scores before the exercise therapy and the achievement rate. The achievement rates of exercise therapy were negatively correlated with SDS-J or SASS-J after exercise therapy in women. The SDS-J after exercise therapy was correlated with neuroticism in men and negatively correlated with extraversion in women. The SASS-J after exercise therapy was negatively correlated with neuroticism and correlated with extraversion and openness in men. In contrast, the SASS-J after exercise therapy correlated with openness and agreeableness in women. Conscientiousness was correlated with the achievement rate of exercise therapy in men, but not with the various personality traits in women.

**Conclusion:**

Depressive symptoms and social adaptation were differently associated with personality traits and achievement rates before and after exercise therapy. Conscientiousness before exercise therapy predicted a higher achievement rate for exercise therapy in men.

## 1. Introduction

Depression is a common psychiatric disorder. Approximately 280 million people worldwide suffer from depression and it affects an estimated 3.8% of the global population (World Health Organization, 2021). Depression is the leading cause of disability worldwide, and accounts for 40.5% of disability-adjusted life years due to psychiatric and substance use disorders (Whiteford et al., 2013). Personality traits also affect physical and mental health (Strickhouser et al., 2017). The etiology of depression is multifactorial, but personality traits are considered one of the most important predictors of depression.

Personality traits are characterized by persistent patterns of thinking, feeling, and behavior that are formed throughout childhood and become increasingly consistent throughout life (Corr and Matthews, 2009). One of the most common conceptualizations of personality is the five-factor personality model, which includes five dimensions of personality traits, including neuroticism, extraversion, openness, agreeableness, and conscientiousness as essential characteristics of personality (McCrae and Costa, 1987). Personality traits may also be influenced by age, gender, and race; for example, neuroticism is known to be higher among women in the American population, but not among Japanese or Black South Africans (Hyde, 2014; Hakulinen et al., 2015; Serrano et al., 2022).

Various studies have investigated the association between personality traits and depression. People with depressive disorders, including major depressive disorder, unipolar depression, and dysthymic disorder, have higher levels of neuroticism and lower levels of extraversion and conscientiousness than healthy controls (Kotov et al., 2010). In a large meta-analysis involving 117,899 participants, low extraversion, high neuroticism, and low conscientiousness were associated with depressive symptoms in cross-sectional analyses. Similar associations were associated with an increased risk of depressive symptoms during follow-ups in longitudinal studies adjusted for baseline depressive symptoms (Hakulinen et al., 2015).

Physical inactivity also increases the risk of depression (Lampinen et al., 2000). A longitudinal retrospective study has suggested that physically active students have a lower incidence of depression (Paffenbarger et al., 1994). Several narrative reviews have concluded that physical activity can prevent depression (Teychenne et al., 2008; Mammen and Faulkner, 2013). We previously reported that an exercise intervention using walking significantly reduced depressive symptoms and improved social adaptation in workers with no exercise habits (Ikenouchi-Sugita et al., 2013). One meta-analysis reported that extraversion and conscientiousness are positively correlated with physical activity, while neuroticism is inversely associated with physical activity (Rhodes and Smith, 2006). Although studies have shown an association between personality traits and depression or physical activity, it is unclear which personality traits are more likely to be associated with adherence and high achievement rates in exercise therapy to prevent major depression. Exercise therapy requires active engagement; therefore, levels of depressive symptoms, social adaptation and personality traits may affect adherence or achievement rates.

The purpose of this study is to determine the effects of personality traits before exercise therapy on depressive symptoms or social adaptation before and after exercise therapy in healthy Japanese workers and to determine the effects of depressive symptoms or social adaptation before and after exercise therapy, and personality traits before exercise therapy on the achievement rate of exercise therapy for the prevention of major depression.

## 2. Materials and methods

### 2.1. Participants and procedures

Participants were recruited among workers at the University of Occupational and Environmental Health, Japan and its affiliated facilities via notice boards at the facilities. The study was explained to 1,529 people, and 257 gave their written consent to participate in the study. Of these, 227 were healthcare workers and 30 were physical laborers. The participants were asked on paper whether they had a history of mental illness. After excluding seven participants with a history of mental illness, 250 participants were assessed for depressive symptoms, social adaptation, and personality traits. Eight weeks of walking were administered as exercise therapy. Thirty-five participants dropped out during the study or gave incomplete information were excluded. The remaining 215 who completed the study were included in the analysis.

The Japanese version of the NEO five-factor inventory (NEO-FFI) was administered as a baseline to assess personality traits before the start of the exercise therapy. The Japanese versions of the Zung self-rating depression scale (SDS-J) (Zung et al., 1965) and social adaptation self-evaluation scale (SASS-J) (Ueda et al., 2011) were used to evaluate participants’ depressive symptoms and social adaptation before and 8 weeks after the start of the exercise therapy.

We analyzed the relationship between the SDS-J or SASS-J before and after exercise therapy and each of the NEO-FFI before exercise therapy; changes in SDS-J and SASS-J before and after exercise therapy; and the relationship between the achievement rate of exercise therapy and the SDS-J or SASS-J before and after exercise therapy and each item of the NEO-FFI before exercise therapy.

### 2.2. Exercise therapy

All the participants received instructions on paper to walk the equivalent of 17.5 kcal/kg/week, spread over at least 3 days per week. This amount of activity is the recommended public health dose and considered effective for mild to moderate depression (Dunn et al., 2005). For example, a participant weighing 60 kg needs to exercise an equivalent of 1,050 kcal per week; if it takes approximately 34 steps to burn 1 kcal, the participant needs to walk 35,700 steps per week. If the participant walks 5 days a week, the goal is 7,140 steps per day. To assess adherence to the specified exercise regimen, participants were instructed to wear a pedometer that displays the number of steps taken and calories burned. The achievement rate (%) was calculated by dividing the number of steps walked by the target number of steps for the 8 weeks and multiplying the results by 100.

### 2.3. Assessment of personality traits

Personality traits were assessed using the Japanese version of the NEO-FFI, which comprises five dimensions: neuroticism, extraversion, openness, agreeableness, and conscientiousness. The Cronbach’s alpha coefficients were 0.83 for neuroticism, 0.78 for extraversion, 0.75 for openness, 0.68 for agreeableness, and 0.77 for conscientiousness, which confirmed the reliability of the Japanese version of the NEO-FFI (Shiranaka et al., 2011).

### 2.4. Assessment of the level of depressive state

The level of depressive symptoms was assessed using the SDS-J, a self-administered 20-item scale. The means (standard deviation) of the raw scores were 35 (12) for normal, 49 (10) for neurosis, and 60 (7) for depression, with SDS-J values increasing in proportion to the depressive state (Zung et al., 1965; Fukuda and Kobayashi, 2011).

### 2.5. Assessment of the level of social adaptation

The level of social adaptation was assessed using the SASS-J, which is a self-administered measure of social adaptation. The higher the SASS-J score, the higher the level of social adaptation. The cutoff value for predicting social adaptation in depressed patients is 25/26. The Cronbach’s alpha coefficient of the SASS-J was 0.81, which confirmed its reliability (Ueda et al., 2011).

### 2.6. Statistical analysis

All the statistical analyses were performed using Stata/SE 17.0. Continuous variable data are presented as means (standard deviation) and nominal variable data as percentages. A univariate analysis of the differences in each item was performed using a paired *t*-test. Statistical data were expressed as standard errors. Univariate correlations for each item were evaluated using Spearman’s rank sum test, and multivariate analyses adjusted for covariates were performed using multiple regression analysis. The test was two-tailed, with a *p*-value < 0.05 considered statistically significant.

## 3. Ethics statement

The study was conducted in accordance with the 1975 Declaration of Helsinki (revised in 2008). All the procedures involving human subjects were approved by the Ethics Committee of the University of Occupational and Environmental Health, Japan (Approval No. 10-076), and written informed consent was obtained from all participants.

## 4. Results

Table 1 summarizes participants’ demographic information. Of the 215 participants, 70 were men, 145 were women, and 197 adhered to the exercise regimen, while 18 did not. The achievement rate of the exercise therapy was 142% for men and 200% for women, which was well above the target number of steps. Table 2 shows the association between personality traits and SDS-J scores at baseline. The SDS-J scores correlated positively with neuroticism and negatively with extraversion, agreeableness, and conscientiousness in men and women. SDS-J and openness showed no association in men but were negatively correlated in women. These trends were similar in both univariate and multivariate analyses. Table 3 shows the associations between personality traits and SASS-J at baseline. The SASS-J was negatively correlated with neuroticism, and positively correlated with extraversion, openness, agreeableness, and conscientiousness in men and women. These trends were similar in both univariate and multivariate analyses. Table 4 shows the changes in SDS-J and SASS-J scores before and after exercise intervention. The SDS-J scores were within the normal range for both men and women, with no significant change before or after exercise therapy. The SASS-J scores were also within the normal range for both men and women, with a significant increase in SASS-J scores after exercise therapy in men, but no significant change in women. Table 5 shows the associations between the baseline SDS-J and SASS-J scores and exercise therapy attainment. No groups showed any association with baseline SDS-J or SASS-J. These trends were similar in both univariate and multivariate analyses. Table 6 shows the associations between the scores of SDS-J or SASS-J at 8 weeks and the achievement rate of exercise therapy. In univariate analysis, no association was found between the achievement rate of exercise therapy and the SDS-J or SASS-J after exercise therapy. Multivariate analysis showed negative associations between the achievement rate of exercise therapy and the SDS-J or SASS-J after exercise therapy in women. Table 7 shows the association between personality traits at baseline and SDS-J at 8 weeks. In univariate analysis, the SDS-J correlated with neuroticism after exercise therapy, while extraversion, openness, and conscientiousness correlated negatively for men and women. The SDS-J after exercise therapy was negatively correlated with agreeableness in women. In multivariate analysis, SDS-J after exercise therapy correlated with neuroticism in men and negatively correlated with extraversion in women. Table 8 shows the association between baseline personality traits and SASS-J scores at 8 weeks. In univariate analyses, the SASS-J after exercise therapy was negatively correlated with neuroticism and positively correlated with extraversion, openness, agreeableness, and conscientiousness for men and women. In multivariate analysis, the SASS-J after exercise therapy showed a similar trend as in univariate analysis for all participants. The SASS-J after exercise therapy was negatively correlated with neuroticism and positively correlated with extraversion and openness in men. The SASS-J after exercise therapy was positively correlated with openness and agreeableness in women. Table 9 shows the association between the achievement rate of the exercise therapy and personality traits. Univariate analysis showed an association between achievement rate and conscientiousness for all groups. The association between achievement rate and extraversion was found only in men. Multivariate analysis showed a positive association between achievement rate and conscientiousness for men.

**Table 1:** Participant demographics.

	All	Men	Women
Participants (*n*)	215	70	145
Age	41.7 (12.4)	41.8 (11.8)	41.7 (12.6)
Exercise habits	63 (29%)	26 (37%)	37 (26%)
**Personality traits**			
Neuroticism	24.7 (7.54)	24.1 (7.96)	25.0 (7.34)
Extraversion	25.1 (6.15)	24.6 (6.57)	25.3 (5.94)
Openness	28.5 (5.37)	27.8 (6.26)	28.9 (4.87)
Agreeableness	31.5 (4.67)	30.8 (4.41)	31.9 (4.76)
Conscientiousness	27.2 (5.68)	26.2 (5.88)	27.7 (5.53)
Adherence	197 (92%)	54 (77%)	143 (99%)
Achievement rate (%)	181 (7.56)	142 (7.15)	200 (6.25)

The continuous variable data are shown as means (standard deviation).

**Table 2:** Relationship between baseline SDS-J and personality traits.

	Univariable		Multivariable			
NEO-FFI	Spearman’s rank correlation coefficient (*r*)	*p*-value	Standard partial regression coefficient (*β*)	Standard error	*t*-value	Adjusted *p*-value
**Neuroticism**						
All participants	0.514	<0.001*	0.586	0.063	10.18	<0.001*
Men	0.506	<0.001*	0.533	0.105	5.14	<0.001*
Women	0.503	<0.001*	0.625	0.079	8.84	<0.001*
**Extraversion**						
All participants	−0.379	<0.001*	−0.420	0.085	−6.63	<0.001*
Men	−0.461	<0.001*	−0.482	0.137	−4.31	<0.001*
Women	−0.356	<0.001*	−0.390	0.108	−5.00	<0.001*
**Openness**						
All participants	−0.217	0.0014*	−0.222	0.104	−3.27	0.001*
Men	−0.226	0.0595	−0.243	0.165	−1.90	0.062
Women	−0.238	0.004*	−0.212	0.139	−2.58	0.011*
**Agreeableness**						
All participants	−0.283	<0.001*	−0.392	0.112	−6.17	<0.001*
Men	−0.305	0.0103*	−0.326	0.213	−2.79	0.007*
Women	−0.321	<0.001*	−0.423	0.135	−5.45	<0.001*
**Conscientiousness**						
All participants	−0.254	<0.001*	−0.271	0.097	−4.06	<0.001*
Men	−0.316	0.0076*	−0.299	0.162	−2.54	0.014*
Women	−0.230	0.0055*	−0.256	0.121	−3.15	0.002*

In the multivariate analysis, the SDS-J was the objective variable, age and exercise habits were covariates, and personality traits were explanatory variables. Sex was also included as a covariate for all participants. *Statistical significance was set at *p* < 0.05.

**Table 3:** Relationship between baseline SASS-J and personality traits.

	Univariable		Multivariable			
NEO-FFI	Spearman’s rank correlation coefficient (*r*)	*p*-value	standard partial regression coefficient (*β*)	standard error	*t*-value	adjusted *p*-value
**Neuroticism**						
All participants	−0.391	<0.001*	−0.462	0.050	−7.39	<0.001*
Men	−0.428	<0.001*	−0.443	0.089	−4.16	<0.001*
Women	−0.374	<0.001*	−0.473	0.062	−6.05	<0.001*
**Extraversion**						
All participants	0.500	<0.001*	0.556	0.057	9.61	<0.001*
Men	0.533	<0.001*	0.584	0.101	5.81	<0.001*
Women	0.488	<0.001*	0.532	0.070	7.47	<0.001*
**Openness**						
All participants	0.373	<0.001*	0.390	0.073	6.09	<0.001*
Men	0.465	<0.001*	0.477	0.120	4.18	<0.001*
Women	0.330	<0.001*	0.349	0.094	4.46	<0.001*
**Agreeableness**						
All participants	0.343	<0.001*	0.387	0.083	6.09	<0.001*
Men	0.398	<0.001*	0.465	0.158	4.39	<0.001*
Women	0.322	<0.001*	0.346	0.098	4.33	<0.001*
**Conscientiousness**						
All participants	0.319	<0.001*	0.371	0.069	5.78	<0.001*
Men	0.486	<0.001*	0.475	0.119	4.51	<0.001*
Women	0.239	<0.001*	0.304	0.084	3.81	<0.001*

In the multivariate analysis, the SASS-J was the objective variable, age and exercise habits were covariates, and personality traits were explanatory variables. Sex was also included as a covariate for all participants. *Statistical significance was set at *p* < 0.05.

**Table 4:** Changes in SDS-J and SASS-J scores before and after exercise intervention.

	Baseline	8 weeks	*p*-value
**SDS-J**			
All participants	37.6 (8.21)	37.0 (6.79)	0.35
Men	36.5 (8.06)	35.6 (8.17)	0.21
Women	38.2 (8.25)	38.1 (8.34)	0.77
**SASS-J**			
All participants	36.8 (6.09)	37.0 (6.79)	0.37
Men	36.6 (6.61)	37.8 (6.80)	0.019*
Women	36.8 (5.85)	36.7 (6.79)	0.73

Paired *t*-tests were used to evaluate the results. *Statistical significance was set at *p* < 0.05.

**Table 5:** Relationship between achievement rate and SDS-J and SASS-J scores at baseline.

	Univariable		Multivariable			
	Spearman’s rank correlation coefficient (*r*)	*p*-value	Standard partial regression coefficient (*β*)	Standard error	*t-*value	Adjusted *p*-value
**SDS-J**						
All participants	0.016	0.82	−0.001	0.588	−0.02	0.99
Men	−0.023	0.85	−0.039	0.885	−0.33	0.74
Women	−0.027	0.75	0.007	0.760	0.09	0.93
**SASS-J**						
All participants	0.026	0.70	0.055	0.793	0.87	0.39
Men	−0.010	0.94	0.013	1.107	0.11	0.91
Women	0.058	0.49	0.087	1.073	1.03	0.31

In the multivariate analysis, the achievement rate was the objective variable, and age, and exercise habits were covariates. SDS-J or SASS-J scores at baseline were explanatory variables. Sex was also included as a covariate for all participants.

**Table 6:** Relationship between achievement rate and SDS-J and SASS-J scores at 8 weeks.

	Univariable		Multivariable			
	Spearman’s rank correlation coefficient (*r*)	*p*-value	Standard partial regression coefficient (*β*)	Standard error	*t-*value	Adjusted *p*-value
**SDS-J**						
All participants	−0.010	0.88	−0.174	0.856	−1.85	0.066
Men	−0.052	0.67	0.105	1.373	0.56	0.58
Women	−0.104	0.21	−0.274	1.077	−2.29	0.023*
**SASS-J**						
All participants	−0.009	0.89	−0.208	1.072	−2.16	0.032*
Men	0.102	0.40	0.010	1.974	0.04	0.97
Women	−0.016	0.85	−0.259	1.311	−2.19	0.030*

In the multivariate analysis, the achievement rate was the objective variable, and age, exercise habits, and SDS-J or SASS-J scores at baseline were covariates. SDS-J or SASS-J scores at 8 weeks were explanatory variables. Sex was also included as a covariate for all participants. *Statistical significance was set at *p* < 0.05.

**Table 7:** Relationship between SDS-J at 8  weeks and personality traits.

	Univariable		Multivariable			
NEO-FFI	Spearman’s rank correlation coefficient (*r*)	*p*-value	Standard partial regression coefficient (*β*)	Standard error	*t*-value	Adjusted *p*-value
**Neuroticism**						
All participants	0.514	<0.001*	0.189	0.062	3.35	0.001*
Men	0.548	<0.001*	0.285	0.089	3.29	0.002*
Women	0.466	<0.001*	0.144	0.085	1.92	0.057
**Extraversion**						
All participants	−0.370	<0.001*	−0.129	0.069	−2.53	0.012*
Men	−0.468	<0.001*	−0.117	0.113	−1.28	0.21
Women	−0.348	<0.001*	−0.131	0.087	−2.11	0.037*
**Openness**						
All participants	−0.217	0.0014*	−0.083	0.074	−1.75	0.082
Men	−0.325	0.0060*	−0.156	0.111	−1.84	0.071
Women	−0.215	0.0096*	−0.061	0.101	−1.04	0.30
**Agreeableness**						
All participants	−0.220	0.0012*	0.008	0.091	0.16	0.88
Men	−0.151	0.21	0.053	0.155	0.63	0.53
Women	−0.313	<0.001*	−0.018	0.114	−0.28	0.78
**Conscientiousness**						
All participants	−0.215	0.0014*	−0.027	0.071	−0.56	0.58
Men	−0.330	0.0052*	−0.099	0.115	−1.20	0.23
Women	0.191	0.021*	0.011	0.091	0.18	0.86

In the multivariate analysis, the SDS-J at 8 weeks was the objective variable, and age, exercise habits, and SDS-J at baseline were covariates. Personality traits were explanatory variables. Sex was also included as a covariate for all participants. *Statistical significance was set at *p* < 0.05.

**Table 8:** Relationship between SASS-J at 8  weeks and personality traits.

	Univariable		Multivariable			
NEO-FFI	Spearman’s rank correlation coefficient (*r*)	*p*-value	Standard partial regression coefficient (*β*)	Standard error	*t*-value	Adjusted *p*-value
**Neuroticism**						
All participants	−0.403	<0.001*	−0.133	0.046	−2.61	0.010*
Men	−0.468	<0.001*	−0.185	0.061	−2.61	0.011*
Women	−0.366	<0.001*	−0.099	0.063	−1.45	0.15
**Extraversion**						
All participants	0.559	<0.001*	0.136	0.060	2.50	0.013*
Men	0.684	<0.001*	0.305	0.078	4.06	<0.001*
Women	0.509	<0.001*	0.057	0.079	0.82	0.41
**Openness**						
All participants	0.423	<0.001*	0.161	0.061	3.33	0.001*
Men	0.478	<0.001*	0.166	0.084	2.14	0.036*
Women	0.407	<0.001*	0.156	0.085	2.56	0.011*
**Agreeableness**						
All participants	0.345	<0.001*	0.111	0.071	2.28	0.024*
Men	0.354	0.0027*	0.031	0.116	0.41	0.68
Women	0.374	<0.001*	0.132	0.089	2.13	0.035*
**Conscientiousness**						
All participants	0.337	<0.001*	0.109	0.058	2.25	0.025*
Men	0.492	<0.001*	0.129	0.086	1.72	0.089
Women	0.288	<0.001*	0.108	0.075	1.78	0.078

In the multivariate analysis, the SASS-J at 8 weeks was the objective variable, and age, exercise habits, and SASS-J at baseline were covariates. Personality traits were explanatory variables. Sex was also included as a covariate for all participants. *Statistical significance was set at *p* < 0.05.

**Table 9:** Relationship between achievement rates and personality traits.

	Univariable		Multivariable			
NEO-FFI	Spearman’s rank correlation coefficient (*r*)	*p*-value	Standard partial regression coefficient (*β*)	Standard error	*t*-value	Adjusted *p*-value
**Neuroticism**						
All participants	0.053	0.44	0.060	0.652	0.92	0.36
Men	0.083	0.50	0.138	0.886	1.17	0.25
Women	0.001	0.99	0.018	0.894	0.21	0.83
**Extraversion**						
All participants	0.129	0.06	0.094	0.788	1.47	0.14
Men	0.247	0.039*	0.194	1.096	1.61	0.11
Women	0.074	0.37	0.069	1.056	0.83	0.41
**Openness**						
All participants	0.078	0.26	0.031	0.907	0.47	0.64
Men	0.042	0.73	−0.032	1.217	−0.25	0.81
Women	0.076	0.36	0.086	1.278	1.04	0.30
**Agreeableness**						
All participants	0.077	0.26	0.074	1.033	1.16	0.25
Men	−0.060	0.62	0.021	1.620	0.18	0.86
Women	0.089	0.28	0.119	1.327	1.43	0.16
**Conscientiousness**						
All participants	0.253	<0.001*	0.158	0.842	2.50	0.013*
Men	0.287	0.016*	0.260	1.177	2.25	0.028*
Women	0.171	0.040*	0.132	1.122	1.61	0.11

In the multivariate analysis, the achievement rate was the objective variable, age and exercise habits were covariates, and personality traits were explanatory variables. Sex was also included as a covariate for all participants. *Statistical significance was set at *p* < 0.05.

## 5. Discussion

This is the first study to clarify the relationship between depressive symptoms, social adaptation, and personality traits in healthy Japanese workers and their impact on the achievement rate of exercise therapy to prevent major depression. This study reveals personality traits in men and women that could make exercise therapy more effective in mentally healthy workers, which will be useful when implementing exercise therapy to prevent major depression in the workplace.

In the present study, both depressive symptoms and social adaptation were within normal limits at baseline. Depressive symptoms were significantly correlated with neuroticism, whereas were negatively correlated with extraversion, agreeableness, and conscientiousness. Depressive symptoms were also negatively correlated with openness only in women.

Previous studies have shown that neuroticism is a predictor of stress, anxiety, and depression, while other factors are protective (Alizadeh et al., 2018). Another report showed that neuroticism is positively correlated with depressive symptoms, whereas extraversion, openness, agreeableness, and conscientiousness are negatively correlated with depressive symptoms (Gong et al., 2020), which is similar to the pre-exercise therapy results of the present study. Data on adults 18 years and older from the 2018 China Family Panel Study showed that conscientiousness, extraversion, and agreeableness are negatively associated with depressive symptoms, whereas openness and neuroticism are positively associated with depressive symptoms (Zhao et al., 2022). In the present study, openness was negatively correlated only in women. Studies have also reported that the negative association between conscientiousness and depressive symptoms is stronger in men than in women, and that the negative association between conscientiousness and agreeableness and depressive symptoms is strongest in older adults, followed by people in the middle-aged group, and then younger groups (Zhao et al., 2022). A prospective cohort study of community residents in Spain aged 35 years and older showed that neuroticism is associated with frequency estimates of subthreshold depressive symptoms; in women, openness is associated with the prevalence of subthreshold depressive symptoms; and in men, high extraversion is a protective factor in the development of subthreshold depressive symptoms. Neuroticism is associated with the prevalence of major depressive episodes in both men and women, and high conscientiousness is a protective factor (Serrano et al., 2022). The results of this study of healthy Japanese workers partially differ from earlier studies on personality traits other than neuroticism, which may have been caused by differences in sex, race, age, and country-specific cultural differences.

The present study’s results show that the level of social adaptation before exercise therapy was negatively correlated with neuroticism, whereas extraversion, openness, agreeableness, and conscientiousness were correlated with social adaptation in both men and women. Previous studies have reported that social and academic adaptation are positively correlated with extraversion and conscientiousness and negatively correlated with neuroticism and depression, mostly consistent with the present study’s results (Klimstra et al., 2018).

In this study, 92% of participants were able to adhere to the exercise therapy, and there was no change in the level of depressive symptoms in either men or women before and after exercise therapy, with a significant improvement in social adaptation in men. The UK government guidelines for adults recommend at least 150 min of moderate-intensity activity per week (Department of Health and Social Care, 2019). According to the Health Survey for England 2016, which included a specific chapter on physical activity, only 66% of men and 58% of women achieved this level (Scholes, 2017). Although strict comparisons could not be made because of the different activity settings, adherence and achievement rates were considered high for this study. We have previously reported that exercise intervention is useful in preventing depression and improving social adaptation in the workplace (Ikenouchi-Sugita et al., 2013). The results of the present study suggest that exercise therapy significantly affects social adaptation in men.

In healthy Japanese workers, the levels of depressive symptoms and social adaptation at baseline had no effect on achievement rates. Studies examining exercise therapy and adherence in patients with major depression have shown that the higher the level of depression, the lower the adherence to exercise (Kruisdijk et al., 2020). This result of the present study may be attributed to the fact that the participants were healthy workers without mental illness. If the subjects had depression or other psychiatric disorders, the depression levels might have affected the achievement rate.

No association between achievement rates and depressive symptoms or social adaptation after exercise therapy was found in the univariate analysis. However, in the multivariate analysis, a negative correlation was found between achievement rates and depressive symptoms or social adaptation after exercise therapy in women. The associations between depressive symptoms or social adaptation and personality traits after exercise therapy were almost similar to the results before exercise therapy in the univariate analysis. In multivariate analysis, the association between depressive symptoms and personality traits after exercise therapy was no longer correlated with neuroticism in women, no longer negatively correlated with extraversion in men, and no longer correlated with openness, agreeableness, or conscientiousness in either sex. After exercise therapy, social adaptation was not associated with neuroticism, extraversion, or conscientiousness in women, and with agreeableness and conscientiousness in men. Baseline depressive state and social adaptation as covariates may have influenced the results.

A correlation between conscientiousness and achievement rate was observed in men but not in women. No other personality traits were associated with achievement rates. The results of the present study show that conscientiousness predicts higher achievement rates in exercise therapy to prevent major depression in men. Individuals with high levels conscientiousness are more effective in developing the incremental behaviors necessary to achieve their goals (Stock and Beste, 2015). Individuals with high conscientiousness are purposeful and have a strong will (Shiranaka et al., 2011). High conscientiousness is also associated with more effective coping strategies that prevent negative life events and stressful experiences and reduce the risk of depression (Weiss et al., 2009). There is an interaction between sex and conscientiousness in the incidence of subthreshold depression, with men reporting a greater protective effect of this personality trait (Serrano et al., 2022). These studies explain the current study’s finding of an association between exercise therapy achievement rates and conscientiousness. In healthy male workers, higher conscientiousness may predict higher achievement rates of exercise therapy and its effects.

Several studies have been published on exercise and personality traits. In a prospective study conducted in Australia, conscientiousness and openness predicted an increase in physical activity, whereas agreeableness was associated with a subsequent decrease in physical activity (Allen et al., 2017a). A study of older adults showed that extraversion, agreeableness, and conscientiousness are positively related to objectively measured physical activity, whereas neuroticism is negatively associated (Artese et al., 2017). A study of female college students also reported a negative relationship between neuroticism and physical activity (Wilson et al., 2015). A meta-analysis has suggested that the main personality traits associated with sedentary behavior are neuroticism (positively associated) and conscientiousness (negatively associated) (Allen et al., 2017b). The association between moderate physical activity and neuroticism, openness, and conscientiousness is known to vary from country to country (Gacek et al., 2021).

The results of the current and previous studies on exercise and personality traits have been inconsistent. These differences may be due to differences in race, sex, cultural background, social position of the workers, and the types and methods of physical activity. This study was conducted using self-administered exercise therapy through paper-based instruction; the results may differ if face-to-face exercise instructions were provided.

Considering the above, one should be cautious in interpreting the present results. One interpretation is depressive symptoms or its associated factors and achievement of exercise therapy may be bidirectional. Depressive state, personality traits, or social adaptation associated with depressive state may influence the achievement of exercise therapy; conversely, exercise therapy may result in behavioral changes in depressive state via depressive state associated personality traits, or social adaptation.

Our study has several limitations. First, because participants volunteered, selection bias could be an issue if nonparticipation is associated with personality traits, various physical activities, or depressive symptoms. Second, adherence rates were high in this exercise therapy setting; therefore, it was impossible to determine the association between adherence and personality traits. Future studies should examine settings in which adherence to exercise therapy can withstand statistical analysis. Third, the presence of a history of mental illness was self-reported on paper. For a more rigorous study, the use of rating scales such as the K6, GHQ-12, and CES-D is preferred. Fourth, personality traits, depressive symptoms, and social adaptation were assessed using self-administered rating scales. Future studies should consider including clinicians to provide more reliable assessments. Fifth, the number of steps was measured using a pedometer that displayed the number of steps and calories burned, but was recorded by filling out a recording form. Future studies using physical activity monitors with accelerometers and long-term recording capabilities are required to evaluate the amount of physical activity. Sixth, although the participants’ exercise habits were confirmed before the start of exercise therapy, the amount of exercise needed to be ascertained, and the possibility of these influences cannot be ruled out. Further studies are required to confirm these findings. Seventh, because the participants were employees of specific worksites and their affiliated worksites in Japan, caution should be exercised when generalizing the present results to workers with different backgrounds. These results should be confirmed in other occupations, industrial areas, and regions. Eighth, preliminary power tests were not conducted. However, these results are considered reliable and statistically significant. Last, we cannot rule out the possibility that other imperceptible confounding factors may have influenced the results. Therefore, further research is needed to confirm and develop these results.

## 6. Conclusion

In this study of healthy Japanese workers, depressive symptoms and social adaptation were differently associated with personality traits and achievement rates before and after exercise therapy. Conscientiousness predicted higher achievement rates for exercise therapy for major depression prevention among healthy Japanese male workers and may be effective in paper-based instruction. Further investigation is needed because this study’s findings cannot be generalized, as age, race, cultural background, workplace, and method of exercise therapy may have influenced the results.

## Data availability statement

The raw data supporting the conclusions of this article will be made available by the authors, without undue reservation.

## Ethics statement

The studies involving human participants were reviewed and approved by Ethics Committee of the University of Occupational and Environmental Health, Japan. The patients/participants provided their written informed consent to participate in this study.

## Author contributions

AI and RY conceived, designed, and conducted the study. AI and NO analyzed the data. AI, NO, TM, and RY took part in drafting, revising, or critically reviewing the article, gave final approval of the version to be published, agreed on the journal to which the article had been submitted, and agreed to be accountable for all aspects of the work. All authors contributed to the article and approved the submitted version.

## Funding

This study was supported and partially funded by a research grant from the University of Occupational and Environmental Health, Japan.

## Conflict of interest

The authors declare that the research was conducted in the absence of any commercial or financial relationships that could be construed as a potential conflict of interest.

## Publisher’s note

All claims expressed in this article are solely those of the authors and do not necessarily represent those of their affiliated organizations, or those of the publisher, the editors and the reviewers. Any product that may be evaluated in this article, or claim that may be made by its manufacturer, is not guaranteed or endorsed by the publisher.
